# Genetic and environmental risk factors for atherosclerosis regulate transcription of phosphatase and actin regulating gene *PHACTR1*

**DOI:** 10.1016/j.atherosclerosis.2016.04.025

**Published:** 2016-07

**Authors:** Michael E. Reschen, Da Lin, Anil Chalisey, Elizabeth J. Soilleux, Christopher A. O’Callaghan

**Affiliations:** a Wellcome Trust Centre for Human Genetics, Nuffield Department of Medicine, University of Oxford, Roosevelt Drive, Oxford, OX3 7BN, United Kingdom; b Nuffield Division of Clinical Laboratory Sciences, Radcliffe Department of Medicine, University of Oxford and Department of Cellular Pathology, John Radcliffe Hospital, Oxford, OX3 9DU, United Kingdom

**Keywords:** Myocardial infarction, Atherosclerosis, Functional genomics, Genetic polymorphism, Genomics, Low-density lipoprotein (LDL), Genetic disease, PHACTR1, Expression quantitative trait locus (eQTL), Oxidized low density lipoprotein (oxLDL), CAD, coronary artery disease, GWAS, genome wide association studies, SNP, single nucleotide polymorphism, PHACTR1, phosphatase and actin regulator 1, oxLDL, oxidized low density lipoprotein, PP1, protein phosphatase 1, PBMC, peripheral blood mononuclear cells, TSS, transcription start site, RACE, rapid amplification of cDNA ends, HAEC, human aortic endothelial cells, LPS, lipopolysaccharide, NLS, nuclear localization signal, DAB, diaminobenzidine

## Abstract

**Background and aims:**

Coronary artery disease (CAD) risk is associated with non-coding genetic variants at the phosphatase and actin regulating protein 1(*PHACTR1*) gene locus. The *PHACTR1* gene encodes an actin-binding protein with phosphatase regulating activity. The mechanism whereby PHACTR1 influences CAD risk is unknown. We hypothesized that *PHACTR1* would be expressed in human cell types relevant to CAD and regulated by atherogenic or genetic factors.

**Methods and results:**

Using immunohistochemistry, we demonstrate that PHACTR1 protein is expressed strongly in human atherosclerotic plaque macrophages, lipid-laden foam cells, adventitial lymphocytes and endothelial cells. Using a combination of genomic analysis and molecular techniques, we demonstrate that PHACTR1 is expressed as multiple previously uncharacterized transcripts in macrophages, foam cells, lymphocytes and endothelial cells. Immunoblotting confirmed a total absence of PHACTR1 in vascular smooth muscle cells. Real-time quantitative PCR showed that *PHACTR1* is regulated by atherogenic and inflammatory stimuli. In aortic endothelial cells, oxLDL and TNF-alpha both upregulated an intermediate length transcript. A short transcript expressed only in immune cells was upregulated in macrophages by oxidized low-density lipoprotein, and oxidized phospholipids but suppressed by lipopolysaccharide or TNF-alpha. In primary human macrophages, we identified a novel expression quantitative trait locus (eQTL) specific for this short transcript, whereby the risk allele at CAD risk SNP rs9349379 is associated with reduced PHACTR1 expression, similar to the effect of an inflammatory stimulus.

**Conclusions:**

Our data demonstrate that *PHACTR1* is a key atherosclerosis candidate gene since it is regulated by atherogenic stimuli in macrophages and endothelial cells and we identify an effect of the genetic risk variant on PHACTR1 expression in macrophages that is similar to that of an inflammatory stimulus.

## Highlights

• PHACTR1 is expressed as two transcripts in both immune and endothelial cells in human atherosclerotic plaque.
• Oxidized-LDL upregulates a short PHACTR1 transcript, but suppresses an intermediate length transcript in macrophages.
• Lipopolysaccharide and TNF-alpha cause the opposite effect with strong suppression of the short transcript in macrophages.
• The coronary artery disease risk SNP, rs9349379, is associated with expression of the short transcript in macrophages.
• The effect of the coronary artery disease risk allele on PHACTR1 mirrors that of inflammatory stimuli.

## Graphical abstract

**Figure:**
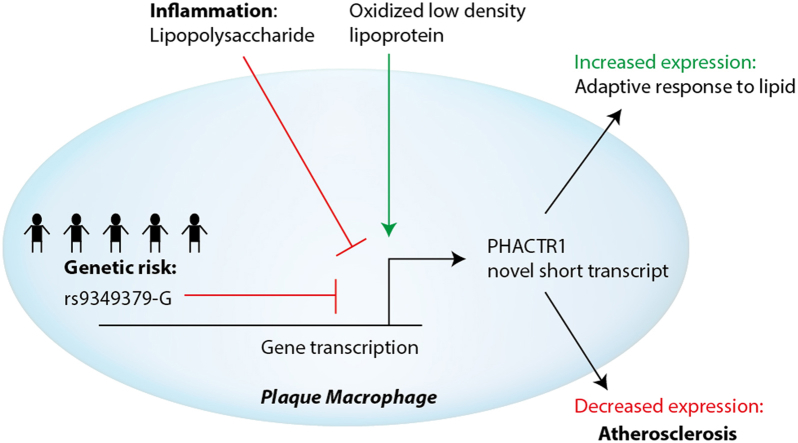

## 1 Introduction

Coronary artery disease (CAD) is caused by atherosclerosis, a form of chronic inflammation. Deposition of pro-inflammatory lipoproteins in arterial walls is associated with the development of atherosclerotic plaques that can obstruct blood flow and trigger the formation of occlusive thrombi [1], [2]. CAD is a complex disease with a strong heritable component [3]. Genome-wide association studies (GWAS) have identified 58 genomic regions where single nucleotide polymorphisms (SNPs) are associated with CAD risk [4], [5]. Most of these disease-associated variants are in non-coding DNA and likely exert their effect predominantly by altering transcription factor binding to regulatory DNA elements [4], [6], [7], [8]. A number of CAD risk loci contain genes that were not previously implicated in coronary artery disease and for which no mechanism has been identified to account for the risk associated with the reported genetic variant. Understanding the effect of these genetic risk variants on the expression and function of the genes that they regulate will advance our understanding of atherosclerosis and could lead to new therapeutic targets.

Several studies have independently replicated an association between CAD and SNPs at a genetic locus containing the phosphatase and actin regulator 1 (*PHACTR1*) gene [9], [10], [11], [12]. SNPs at the *PHACTR1* locus are associated with the specific phenotypes of early onset myocardial infarction, coronary artery calcification [11], [12] and with an intermediate phenotype of impaired central hemodynamic indices, indicating abnormal vascular stiffness [13]. The *PHACTR1* locus is pleiotropic since the protective alleles of the CAD risk SNPs are associated with an increased risk of ischemic stroke caused by cervical artery dissection, a form of non-atherosclerotic vascular disease [14]. The variants reported in these studies lie in an intronic region of *PHACTR1* over 250 kb away from any other gene. A genetic fine mapping study suggested that rs9349379 was the most likely causative variant at the locus and it was associated with expression of *PHACTR1* mRNA in composite right coronary artery tissue [15]. An expression quantitative trait locus (eQTL) study assaying gene expression in diverse human tissues showed that rs9349379 also affected *PHACTR1* mRNA expression in aortic artery and tibial artery tissue [16]. In a whole genome epigenetic and expression study, we recently demonstrated that *PHACTR1* is one of the most highly upregulated genes in macrophages exposed to oxLDL [17].

The *PHACTR* family consists of 4 genes encoding proteins that interact directly with both actin and protein phosphatase 1 (PP1) [18]. *PHACTR1* was originally cloned from a rat brain cDNA library using a yeast two-hybrid system with PP1 as bait [18]. The human *PHACTR1* gene is on chromosome 6 and a transcript with a 1743 bp open reading frame has been cloned from human brain [19]. The resulting 580 amino acid protein has 4 highly conserved actin-binding RPEL domains and both mouse and rat orthologues have been shown to bind actin [18], [20]. Human PHACTR1 protein contains nuclear localization signal (NLS) motifs at both ends of the protein that, in the mouse ortholog, have been shown to facilitate importin-dependent nuclear translocation in response to serum stimulation [20]. Binding of the RPEL domains to G-actin maintains PHACTR1 in a cytoplasmic location [20]. Serum induces Rho-dependent remodeling of actin from G- to F-actin and translocation of PHACTR1 into the nucleus [20].

PP1 is part of a family of serine/threonine phosphatases that are present in both the nucleus and cytoplasm [21]. The enzymatic specificity of PP1 enzymes is achieved in part by their association with accessory proteins [22]. PHACTR1 may function as an accessory protein since PHACTR1 binds to PP1 and inhibits its activity *in vitro* [18]. PHACTR1 may also affect actin structure as siRNA-mediated PHACTR1 knockdown in human umbilical vein endothelial cells (HUVEC) reduces F-actin filament numbers and repartitioning as well as increasing cell protrusion dynamics [23]. Although the precise cellular role of PHACTR1 remains to be determined, overexpression and siRNA-mediated knockdown experiments suggest a role in cell motility and vascular morphogenesis [20], [23], [24].

Atherosclerosis involves the interplay of genetic factors with atherogenic stimuli, such as modified low-density lipoprotein, in cell types including macrophages, lymphocytes, endothelial cells and vascular smooth muscle cells [1], [25], [26], [27]. The role of PHACTR1 in these cells and the mechanism whereby it alters CAD risk are unknown. Understanding the role of PHACTR1 is complicated by the fact that the human *PHACTR1* gene is predicted to encode multiple transcripts that have not been characterized. In this study, we hypothesized that PHACTR1 is regulated by atherogenic or inflammatory stimuli and that its expression is influenced by CAD-associated genetic variants. After establishing the expression of PHACTR1 protein in atherosclerotic lesions and the profile of human *PHACTR1* transcripts in primary cell types involved in atherosclerosis, we determined the responses of these transcripts to inflammatory stimuli and to atherogenic lipid. The role of PHACTR1 in CAD was highlighted by its *in vivo* abundance in macrophages and foam cells in human atherosclerotic plaque. The CAD risk allele at SNP rs9349379 was associated with significantly reduced expression of a short transcript in macrophages such that the risk genotype mirrors the effect of an inflammatory stimulus.

## 2 Materials and methods

### 2.1 Ethical approval

Ethical approval for the study was obtained from the NHS Research Ethics Committee (South Central-Hampshire B, reference 13/SC/0392) and all participants provided written informed consent.

### 2.2 Cell isolation, culture and reagents

Primary human coronary artery vascular smooth muscle cells (Invitrogen, Carlsbad, CA) were cultured in Medium 231 (Invitrogen) and Smooth Muscle Growth Supplement (Invitrogen). Primary human aortic endothelial cells (Invitrogen) were cultured in Medium 200 (Invitrogen) with Low Serum Growth Supplement. CD14^+^ monocytes, T cells (CD3^+^) and B cells (CD19^+^) were isolated from the peripheral blood of healthy human volunteers. Blood was centrifuged over Ficoll-Paque PLUS (GE Healthcare LifeSciences, Buckinghamshire, UK) to isolate peripheral blood mononuclear cells (PBMCs). Subsets of cells were isolated using positive selection with magnetic bead-conjugated antibodies (Miltenyi Biotec, Bergisch Gladback, Germany). Individual populations were confirmed to be >95% pure using flow cytometry for the markers used for positive selection. The cells remaining after CD14^+^ cells were magnetically separated from PBMCs were termed CD14^+^-depleted PBMCs and constitute a mixed predominantly lymphocytic population. CD14^+^ monocytes were differentiated into macrophages by 7 days of culture in RPMI 1640 medium with 10% fetal calf serum, 4 mM l-glutamine, 50 units/ml penicillin and 50 mcg/ml streptomycin (Sigma, St Louis, MO), supplemented with 50 ng/ml macrophage colony stimulating factor (M-CSF, eBioscience, San Diego, CA). Foam cells were generated by treating macrophages with human oxLDL (50 mcg/ml) for 48 h. Foam cell formation was confirmed by oil red O staining for intracellular neutral lipid by light microscopy. Macrophage and foam cell viability was shown to be >98% using the Invitrogen LIVE/DEAD microscopy kit.

Ultracentrifugation of freshly isolated human plasma using a discontinuous potassium bromide gradient was used to purify LDL with density 1.019–1.063 g/ml [28]. LDL was extensively dialyzed against PBS, and incubated with 25 μM CuCl_2_ at 37° for 18 h to produce oxidized-LDL. To terminate oxidation 1 mM EDTA was added and oxLDL was stored at 4°. The TBARS assay was used to confirm that LDL was oxidized (Caymen Chemical, Ann Arbor, Michigan). Careful precautions were taken to ensure low endotoxin levels and LDL was tested for endotoxin using the gel clot method after heating to 75° for 15 min to remove the plasma inhibitor (Associates of Cape Cod, East Falmouth, MA). LDL was used at levels of <0.1 EU/ml.

### 2.3 Genotyping of rs9349379

Individuals providing blood samples were genotyped by the Oxford Biobank and additional primary cells were genotyped using an inventoried Taqman genotyping assay according to the manufacturer’s instructions (Applied Biosystems, Foster City, CA, ID 4351379).

### 2.4 RNA extraction and cDNA synthesis and 5′ RACE

RNA was extracted using the Trizol RNA Plus extraction kit with on-column DNAse treatment according to the manufacturer’s instructions (Invitrogen). cDNA was produced by reverse transcription of 1 mcg of RNA using Bioscript reverse transcriptase (Bioline, London, UK) with random hexamers (Qiagen, Venlow, Limburg, Netherlands). For 5′ RACE, 5 mcg of total RNA was reverse transcribed using 20 pmol of gene specific primer (CO5441 TTTGGCTGAAGGATCTTGGG) and then poly A tailed at the 3′ cDNA end. First round PCR was performed using an inner gene specific primer (CO5439 CCATCCATGATGTCTGACGGTTGG) and oligo(dT) primer (with additional 5′ sequence for round two PCR, CO1352) for 18 cycles using Bioscript (Bioline). Second round PCR was performed using gene specific primer (CO5400 CTTCTCTGCTTTGCCTCATAGATATTT) and a primer (CO3984 CTAGGAATTCTAGAGGTACCTCGAG) annealing to a 5′ prime site created in the 1st round PCR. PCR products were gel extracted and ligated into Strataclone vector pSC-A-amp/kan using the strataclone PCR cloning kit (Agilent, Santa Clara, CA). Colonies were cultured, plasmid DNA extracted using a miniprep kit and sequenced using BigDye (Invitrogen) with both inner gene-specific primer (CO5513 CTGACGTGTGTTTGAACTTTTCGC) and also (CO5400 CTTCTCTGCTTTGCCTCATAGATATTT). Sequences were aligned to the hg19 reference genome using the BLAT program in the UCSC genome browser.

### 2.5 *PHACTR1* cloning

The long *PHACTR1* transcript was amplified from an Image clone (IRATp970E05120D) using primers CO4400 ACTGATGATCAATGGATTATCCCAAAATGGATTATTTTC, and CO4807 TCAGTCTCGAGTTAAGGTCGGTGAAACCTTGTTAAGTG and cloned into pCDNA3.1 with an N-terminal myc tag to produce plasmid pOC1259. The intermediate transcript was amplified from CD14^+^ macrophage cDNA using primers CO4976 ACTGATGATCAATGCGTTCTGACTCCCTCGTCC and CO4807 and cloned into pCDNA3.1 with an N-terminal myc tag to produce plasmid pOC1256. The short *PHACTR1* transcript (ENST00000379335) was amplified from macrophage cDNA using CO4806 AATGAGGATCCATGTATCTGCAAGGGCCGAGG and CO4807 and cloned into pCDNA3.1 with an N-terminal Myc tag to make plasmid pOC1257. Plasmids were sequenced using BigDye.

### 2.6 Tissue bank and primary cell expression analysis

An oligo(dT)-primed cDNA library set made from adult and fetal human tissue was screened for expression of *PHACTR1* transcripts. *PHACTR1* transcripts and beta-actin controls were amplified using BioTaq polymerase with 33 cycles and 25 cycles respectively. Products were run on a 1% agarose gel and stained using ethidium bromide. The primer pairs used were as follows: long transcript, CO3948 TGCTCACAGACTCTTGGATGTTG and CO3880 CTTCTCTGCTTTGCCTCATAGATATTT; intermediate transcript, CO3879 GGAAGAAGAAAAGCGAAAAGTTCA and CO3876 GGCAGTAGCAGGACTGGTTTG; short transcript, CO4987 ATGTATCTGCAAGGGCCGAG and CO3944 TGAATTCATTGAGCTCCTTTCG; control beta-actin, CO1851 TCCAGCCTTCCTTCCTGGGCAT and CO1852 GTCAAGAAAGGGTGTAACGCAACT.

### 2.7 Quantitative PCR and eQTL analysis

Expression levels of *PHACTR1* were determined by RT-qPCR with SYBR green reagents (Invitrogen) using either cDNA from macrophages or CD14^+^-depleted PBMCs from genotyped healthy individuals from the Oxford Biobank. RT-qPCR primers used were: CO5399 GGAAGAAGAAAAGCGAAAAGTTCA and CO5400 CTTCTCTGCTTTGCCTCATAGATATTT (intermediate transcripts); CO4987 ATGTATCTGCAAGGGCCGAG and CO5122 CTCTTGATCTCTCTCTTCTCCTCCTG (short transcript); CO3744 TTGCCATCAATGACCCCTTCA and CO3745 CGCCCCACTTGATTTTGGA (control *GAPDH*). Melt curve analysis demonstrated a single product for each primer pair. For each sample, triplicates were assayed. A Kruskal-Wallis test was applied across the three possible genotypes to identify a significant genotype-expression relationship with significance defined as a *p* value < 0.05. To assess *PHACTR1* expression in response to stimuli, primary human macrophages (rs9349379 AA) were treated with 50 mcg/ml oxLDL for 48 h, 10 ng/ml LPS (Sigma) for 24 h, 10 ng/ml TNFα for 24 h (eBioscience), 30 mcg/ml OxPAPC (Invivogen, San Diego, CA) for 48 h, or 10 μg/ml cyclodextrin-cholesterol (Sigma) for 48 h and assayed in biological triplicates. HAEC (rs9349379 AG) were treated similarly with 50 μg/ml oxLDL for 48 h, 10 ng/ml TNFα or 10 μg/ml cyclodextrin-cholesterol for 48 h. Coronary artery smooth muscle cells (rs9349379 AG) were treated with 10 μg/ml cyclodextrin-cholesterol for 48 h and assayed in triplicate. Student’s t-test was applied with significance defined as a *p* value < 0.05.

### 2.8 Western blotting

Cells were lyzed in RIPA buffer with protease inhibitors and subjected to SDS-PAGE analysis using 10 mcg of protein per lane and PageRuler Plus pre-stained marker lane. Endogenous PHACTR1 was detected using a rabbit polyclonal anti-PHACTR1 antibody (Novus Biologicals, Littleton, CO) and secondary HRP-conjugated goat anti-rabbit IgG antibody (Vector labs, Burlingame, CA). Chemiluminescent reactions were performed using ECL Advance (GE Healthcare LifeSciences) and imaged using either a BioRAD gel doc imaging station (BioRad, Hercules, CA) or Amersham hyperfilm ECL (GE Healthcare LifeSciences).

### 2.9 Immunohistochemistry

Formalin-fixed paraffin-embedded tissue samples of non-atherosclerotic aorta (n = 5), and atherosclerotic aorta (n = 5), obtained with full ethical approval from the National Research and Ethics Service (Oxfordshire Research and Ethics Committee A: reference 04/Q1604/21), were immunostained for PHACTR1 with a rabbit polyclonal anti-PHACTR1 antibody (Abcam, Cambridge, UK) detected with the NovolinkTM max polymer detection system (Leica Microsystems, Buffalo Grove, IL), as *per* the manufacturer’s instructions, or with detection reagents only, as a negative control. Slides were mounted in Aquatex mounting medium (Merck, White House Station, NJ). Stained sections were photographed with a Nikon DS-FI1 camera with a Nikon DS-L2 control unit (Nikon, Tokyo, Japan) and an Olympus BX40 microscope (Olympus, Tokyo, Japan). All immunohistochemical staining was analyzed by and performed under the supervision of an experienced board certified consultant pathologist (EJS).

## 3 Results

### 3.1 *PHACTR1* expression is increased in human atherosclerotic lesions

**Fig. 1:**
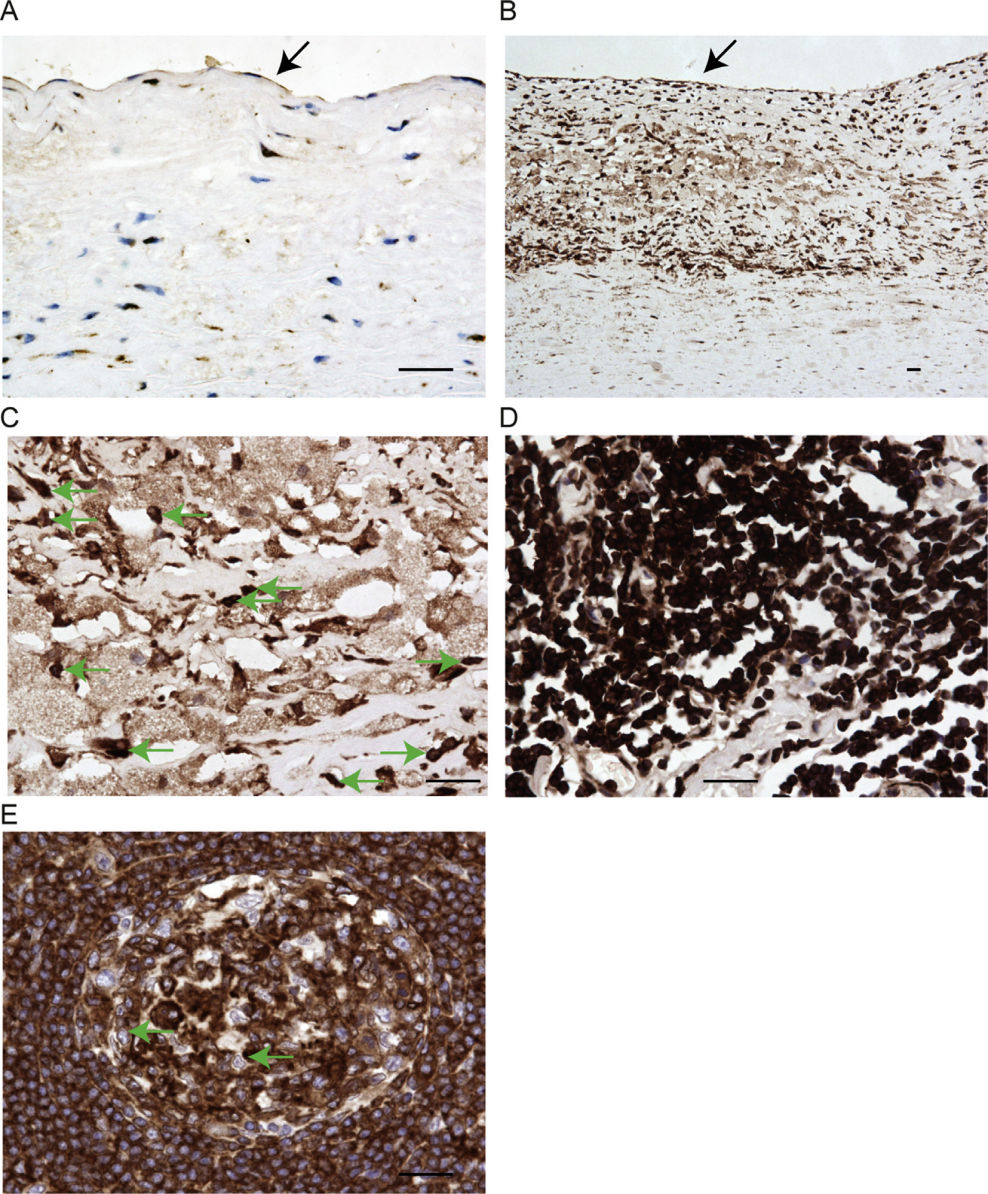
**Immunohistochemistry demonstrates *in vivo* PHACTR1 expression in atherosclerotic plaque**. (A) Healthy aorta demonstrates PHACTR1-positive endothelium (arrowed), but vascular smooth muscle cells in the sub-intimal layer lack PHACTR1 staining. (B) Atherosclerotic plaque showing endothelial positivity (arrowed) and positivity in the sub-intimal space, comprising predominantly foam cells. (C) Higher power view of foam cells in atherosclerotic plaque showing cytoplasmic and nuclear staining for PHACTR1 (Green arrows indicate examples of strongly stained foam cell nuclei. Cytoplasmic foam cell staining is present in the lighter brown areas that asymmetrically encircle the nuclei). (D) Lymphocytic aggregate in aortic adventitia of atherosclerotic vessel showing strong positivity for PHACTR1. The PHACTR1 staining is present throughout the cells, indicating nuclear and cytoplasmic distribution. (E) Germinal centre in tonsillar lymphoid tissue; lymphocytes lack nuclear PHACTR1 as demonstrated by a thin rim of brown cytoplasmic staining with unstained, blue nuclei (example lymphocytes arrowed). Staining for human PHACTR1, using HRP/DAB, each section representative of data from 5 individuals per condition, scale bars indicate 50 μm.

To investigate the role of PHACTR1 in CAD, we used immunohistochemistry to evaluate human PHACTR1 protein expression *in vivo* in atherosclerotic lesions in arteries from patients with atherosclerosis (n = 5) and in normal arteries from healthy controls (n = 5). In healthy arteries, PHACTR1 was expressed in endothelial cells and in the occasional immune cells that were present, but not in vascular smooth muscle cells (Fig. 1A). In atherosclerotic plaque lesions, an abundance of infiltrating macrophages and foam cells were present that all strongly expressed PHACTR1, but no expression was seen in vascular smooth muscle cells (Fig. 1B, C). Plaque endothelial cells also expressed PHACTR1 (Fig. 1B). Lymphocytes in peri-plaque adventitial aggregates also expressed PHACTR1 and this was present in both the cytoplasm and nucleus (Fig. 1D). PHACTR1 was also expressed by lymphocytes in secondary lymphoid tissue (tonsillar), but here it was predominantly in the cytoplasm, rather than the nucleus (Fig. 1E). Overall, the immunohistochemistry results highlight the marked expression of PHACTR1 in immune cells in atherosclerosis, particularly in macrophages and lipid-laden foam cells.

### 3.2 *PHACTR1* expresses multiple transcripts from two promoters in vascular and immune cells

**Fig. 2:**
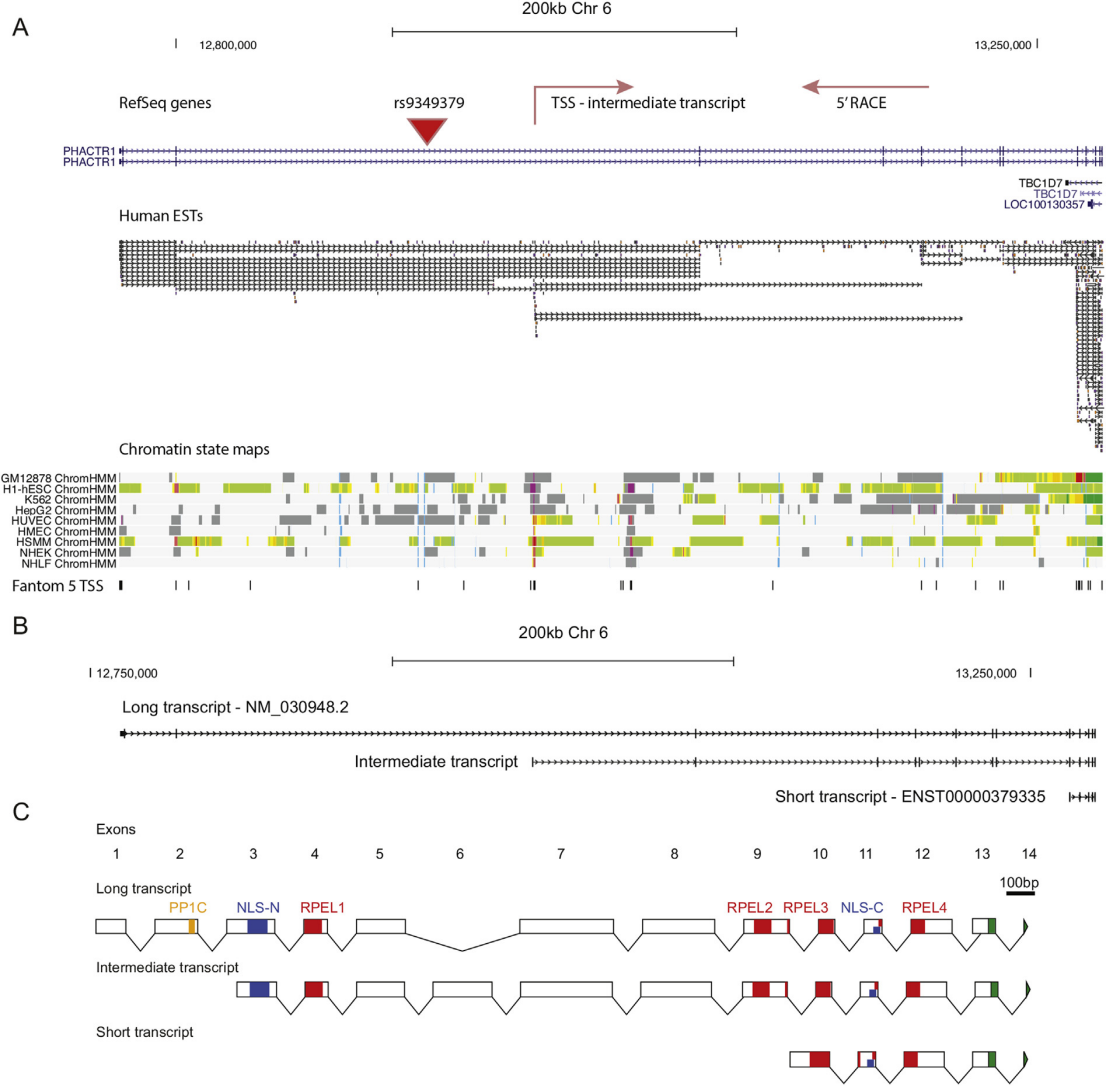
**The structure of human *PHACTR1* transcripts**. (A) The transcript structure for *PHACTR1* is shown for RefSeq curated transcripts showing 2 transcripts in blue. Shown below is human expressed sequence tag data, which is suggestive of a transcription start site halfway along the gene and a further possible site at the 3′ end of the gene. These data were compared to chromatin state data that uses multiple ChIP-seq markers to classify the genome into different types of regulatory element or transcription state. Chromatin state maps are shown for 9 cell types with the chromatin state colored (red - promoter, orange - enhancer, yellow - weak enhancer, blue - insulator, green - transcribed, dark grey - not transcribed). Red regions depicting active promoter elements are seen half way along the gene, coinciding with a TSS suggested by EST data. In one cell type, a promoter is also seen at the 3′ end. These sites coincide with precise TSSs identified by CAGE-seq in a variety of human cell types. Taken together, these data suggest three main TSS in three main promoter regions. The position of coronary artery disease risk variant, rs9349379, is indicated by the red triangle. (B) The transcript structure for the RefSeq transcript is shown uppermost and is aligned to the intermediate transcript that was cloned from macrophages and endothelial cells. The short transcript cloned from macrophages is also shown below. Vertical lines indicate exons. (C) Coding structure of PHACTR1 transcripts with exons drawn to scale and motifs annotated. Motif locations were predicted using the Eukaryotic Linear Motif resource. The region of experimentally proven PP1 binding is shown in green.

We next determined the expression pattern of *PHACTR1* in primary human atherosclerosis-related cell types involved in atherosclerosis. A combined genomic analysis of human expressed sequence tag (EST) data, chromatin state mapping based on ChIP-seq data and CAP analysis of gene expression with high throughput sequencing (CAGE-seq) [29], [30] suggested the presence of a transcriptional start site (TSS) around 240 kb downstream from that of the reported Refseq transcript (NM_030948.2) and a further TSS close to the 3′ end of this transcript (Fig. 2A). To determine the origin of transcripts in cell types involved in atherosclerosis, we mapped the TSS using Rapid Amplification of cDNA Ends (RACE) in primary human macrophages, aortic endothelial cells (HAEC), monocytes and peripheral blood mononuclear cells (PBMC) depleted of CD14^+^ monocytes (Fig. 2A, Supplementary data 1). This demonstrated that the known TSS of the human RefSeq transcript (NM_030948.2) was not used, but instead the RACE clones confirmed a TSS 240 kb downstream, that was supported by the EST, chromatin state and CAGE-seq data (Fig. 2A). We used the RACE results to design primers to amplify and clone a 1674 bp open reading frame from primary human macrophages and arterial endothelial cells. This transcript encodes a 557 amino acid PHACTR1 isoform that matches the predicted NCBI Reference Sequence: XP_011512694.1 (Fig. 2A/B, Supplementary data 1). On the basis of its length, we refer to this transcript as the ‘intermediate transcript’. The open reading frame of the intermediate transcript begins partway through exon 3 of NM_030948.2 and contains all subsequent downstream exons. Additionally, the intermediate transcript includes an extra exon (exon 6) that is not present in NM_030948.2 (Fig. 2B/C). In CD14^+^-depleted PBMCs, the transcriptional start site was around 600 bp upstream of that seen in both primary arterial endothelial cells and CD14^+^ cells, but this did not alter the open reading frame.

The genomic analysis supported the presence of a distal TSS that matched the beginning of ENSEMBL transcript ENST00000379335 [29], [30] (Fig. 2 A/B). Additional analysis of chromatin mapping data from our previous study of primary human macrophages demonstrated an open chromatin site at the TSS that was flanked by H3K27ac-marked histones consistent with promoter activity [17]. Using this information, we cloned a 435 bp transcript from macrophages, which encoded a 144 amino acid protein. This ‘short’ transcript shares its last four 4 exons with the longer transcripts, but begins with an extended version of exon 10 that starts 5′ to the exon 10 start position seen in the longer transcripts (Fig. 2B/C).

### 3.3 PHACTR1 transcripts display tissue-specific differential expression

**Fig. 3:**
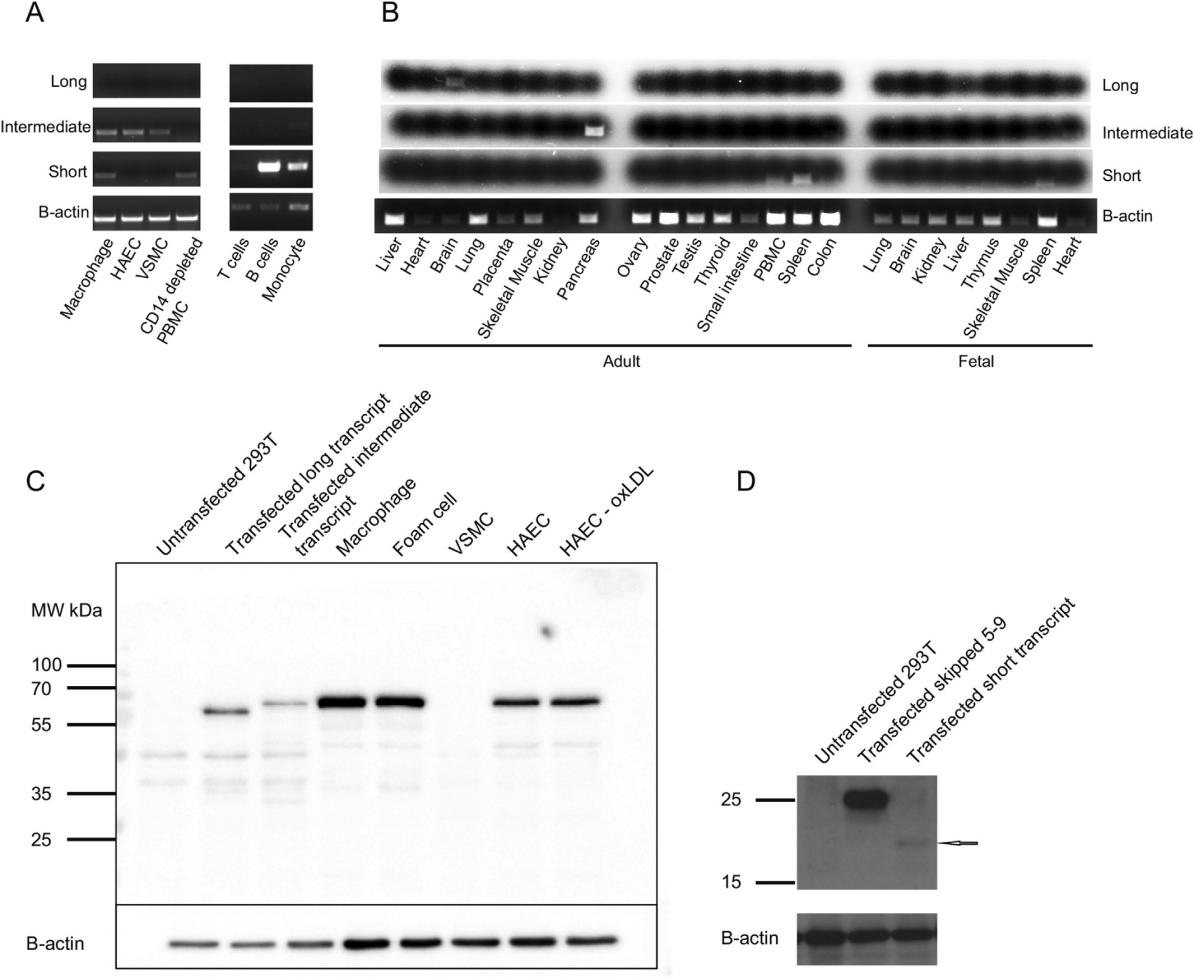
**PHACTR1 expression patterns in primary cells and tissues**. (A) Expression in purified primary human cells showed that the long transcript was not expressed among the cells tested. By contrast, the intermediate transcript showed robust expression in human monocytes, macrophages, arterial endothelial cells (HAEC) and vascular smooth muscle cells (VSMC), but was not expressed by T cells and was barely detectable in B cells and PBMC-depleted of monocytes. (B) Analysis of a human tissue cDNA panel showed the long transcript exclusively expressed in adult brain. The intermediate transcript showed strong expression in the pancreas, but was also detectable in fetal heart. By contrast, the short transcript was present in PBMC and spleen, including fetal spleen. (C) Western blotting: PHACTR1 over-expression in 293T cells transfected with the long and intermediate isoforms compared to endogenous PHACTR1 in primary human cells. The shorter intermediate isoform has an apparently greater size than the long isoform in 293T cells. Macrophages, foam cells and HAECs expressed PHACTR1 with a similar size to the intermediate isoform. PHACTR1 protein was absent from VSMC. A band corresponding to endogenous expression of the short isoform was not seen in any cell type. The polyclonal rabbit anti-PHACTR1 antibody identifies a non-specific additional band at about 47 kDa even in untransfected cells, which do not express PHACTR1. (D) Western blotting with over-expression in 293T cells of the short transcript cloned from macrophages. A cloned form of the intermediate transcript skipping exons 5–9 is also shown for comparison.

We designed transcript-specific primer pairs to examine the expression pattern of the long, intermediate and short transcripts in atherosclerosis-related cell types (Table 1). The long transcript was not expressed in primary human monocytes, macrophages, HAEC, VSMC, T cells, B cells or CD14^+^-depleted PBMC (Fig. 3A). The intermediate transcript was expressed in macrophages, HAEC and VSMC (Fig. 3A), but was barely detectable in monocytes and B cells and absent from T cells. The short transcript was expressed in primary human monocytes, macrophages, B cells, CD14^+^-depleted PBMC, and to a lesser extent in T cells, but was absent from HAEC and VSMC (Fig. 3A).

We screened a panel of adult and fetal tissues and the long transcript was only present in adult brain tissue (Fig. 3B). The intermediate transcript was expressed in pancreas and fetal heart (Fig. 3B). The short transcript was expressed in adult spleen, fetal spleen, PBMC, colon and at a low level in the thymus (Fig. 3B). Overall, the long transcript is restricted to neuronal tissue only and the intermediate transcript is more variable, being present in HAEC, VSMC and macrophages. The short transcript is restricted to immune cells.

### 3.4 PHACTR1 protein is expressed in macrophages and HAEC but not VSMC

To assess PHACTR1 protein expression in primary human cells, western blotting was undertaken using an antibody raised against a region of the protein shared by all PHACTR1 isoforms. Recombinant PHACTR1 isoforms were expressed and analyzed in parallel to facilitate molecular size comparison (Fig. 3C). The isoform encoded by the long transcript migrated with an apparent molecular weight of about 75 kDa (predicted 66 kDa, pI 6.51), similar to that of the rat brain isoform (Fig. 3C) [18]. The intermediate isoform is 23 amino acids shorter, but migrated with an apparently higher molecular weight, equivalent to about 80 kDa (predicted 64 kDa, pI 6.81) (Fig. 3C). This apparently anomalous migration pattern may arise from differences between the protein isoforms such as shape, rigidity, charge or post translational modification. Notably, exon 6 is rich in proline residues that may increase rigidity (14 of 69 residues in exon 6 are proline). Exon 6 in the intermediate isoform does not contain any additional predicted glycosylation motifs. PHACTR1 protein was completely absent from primary vascular smooth muscle cells despite moderate level mRNA expression in these cells (Fig. 3C). In primary human macrophages, foam cells, HAEC and oxLDL-treated HAEC there was strong expression of the intermediate isoform (Fig. 3C). We did not detect protein expression of the short transcript but detection of this protein was difficult even following overexpression in 293T cells (Fig. 3C and D). Although it was not possible to assess the endogenous short isoform reliably, these findings are consistent with our transcript studies and indicate that primary macrophages and arterial endothelial cells express the intermediate, but not the long protein isoform.

### 3.5 PHACTR1 expression is regulated by oxLDL and inflammatory stimuli in macrophages and endothelial cells

**Fig. 4:**
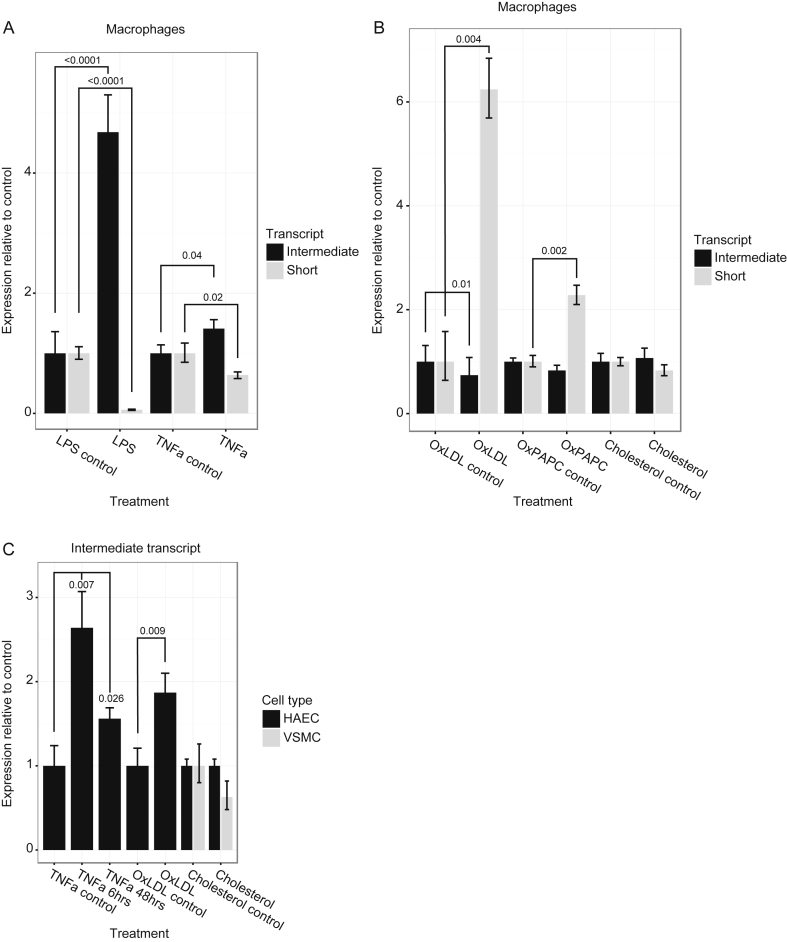
*PHACTR1* transcripts are differentially regulated by atherogenic and inflammatory stimuli. (A) Effect of LPS (10 ng/ml) on mRNA expression in primary human macrophages. The intermediate transcript was upregulated, but the short transcript was suppressed. TNFα (10 ng/ml) produced a directionally similar effect. (B) Exposure of macrophages to oxLDL (50 mcg/ml) caused modest downregulation of the intermediate transcript and strong upregulation of the short transcript. OxPAPC (30 mcg/ml) caused a directionally similar effect to oxLDL on *PHACTR1* expression. (C) The intermediate transcript was upregulated by oxLDL in aortic endothelial cells. TNFα (10 ng/ml) caused upregulation of PHACTR1 at 6 and 48-h time points in aortic endothelial cells. The short transcript was not expressed in aortic endothelial cells. Cyclodextrin-cholesterol (10 mcg/ml) loading of endothelial cells and vascular smooth muscle cells had no effect on PHACTR1 expression. Significant differences are indicated by black lines with *p* values shown. The controls labelled on the x-axis represent the relevant buffer controls for each treatment.

Given the association of the *PHACTR1* locus with CAD and its expression in atherosclerotic lesions, we hypothesized that PHACTR1 would be regulated by atherogenic and inflammatory stimuli. We used transcript-specific RTqPCR to assess expression of PHACTR1 in response to a variety of stimuli. Lipopolysaccharide (LPS) treatment of primary human macrophages upregulated the intermediate transcript (fold = 4.68, *p* < 0.0001), but downregulated the short transcript more than 10-fold (fold = 0.06. *p* < 0.0001) (Fig. 4A). The inflammatory cytokine Tumor Necrosis Factor-alpha (TNFα) produced a similar, but weaker effect with upregulation of the intermediate transcript (fold = 1.41, *p* = 0.04) and downregulation of the short transcript (fold = 0.64, *p* = 0.02) (Fig. 4A). OxLDL exerted a converse effect on expression of PHACTR1 compared to LPS and TNFα; the short transcript was upregulated 6 fold (*p* = 0.004) and the intermediate transcript was downregulated (fold = 0.74, *p* = 0.01) (Fig. 4B). Oxidized 1-palmitoyl-2-arachidonyl-sn- glycero-3-phosphorylcholine (oxPAPC) is a mixture of oxidized phospholipids that have a similar biological effect to minimally oxidized LDL [31]. OxPAPC contains components of the biologically active fractions of HPLC-separated minimally-modified LDL [31]. In macrophages OxPAPC produced a similar directional effect to oxLDL—upregulation of the short transcript (fold = 2.28, *p* = 0.002) and a trend towards downregulation of the intermediate transcript (fold = 0.83, *p* = 0.12) (Fig. 4B). To refine the lipid species responsible for the effect on PHACTR1 expression we loaded macrophages with cholesterol itself. Cells were treated with a cyclodextrin-cholesterol complex that promotes cholesterol loading of macrophages and vascular smooth muscle cells [32], [33]. Unlike the oxidized stimuli, cholesterol loading had no effect on PHACTR1 expression (Fig. 4B). Overall, these data indicate that in macrophages inflammatory pathways downregulate the short transcript and upregulate the intermediate transcript, whereas oxidized lipid-responsive pathways upregulate the short transcript. In human aortic endothelial cells—which only express the intermediate transcript—both TNFα and oxLDL upregulated PHACTR1 (fold = 2.5, *p* = 0.007, and fold = 1.8, *p* = 0.009, respectively) (Fig. 4C). As with macrophages, cholesterol loading with cyclodextrin-cholesterol had no effect on PHACTR1 expression. In vascular smooth muscle cells, cholesterol loading had no effect on expression of the intermediate *PHACTR1* transcript.

### 3.6 Genetic regulation of PHACTR1 expression by CAD SNP rs9349379

**Fig. 5:**
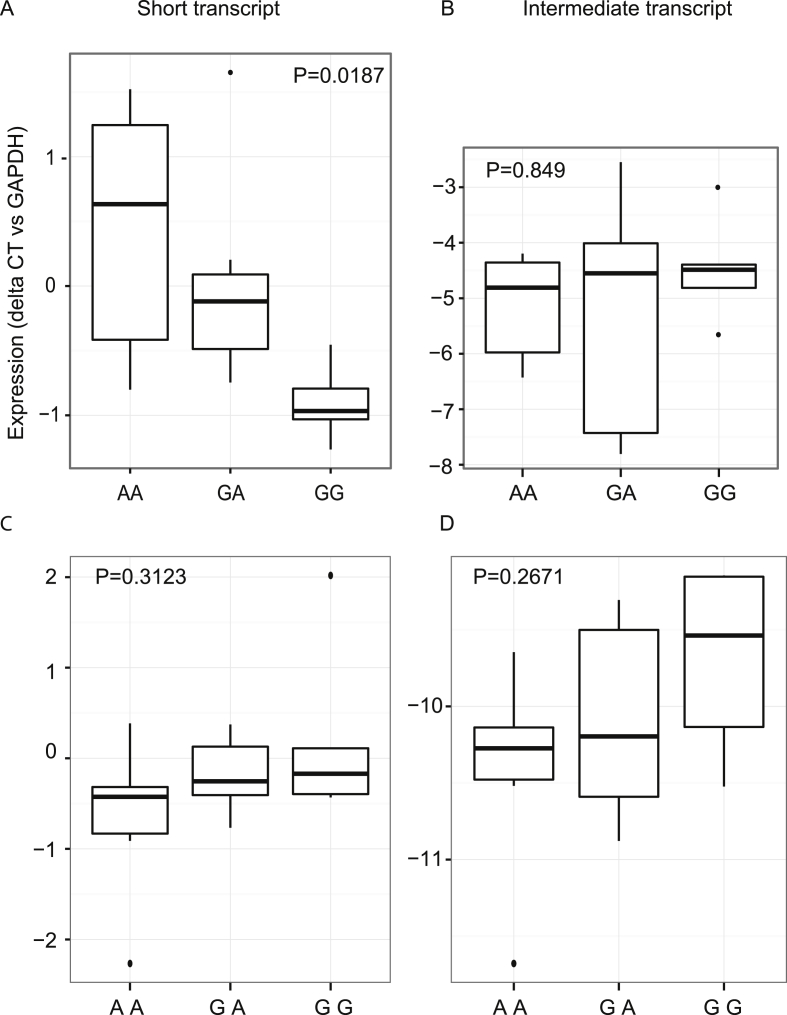
**Genotype of CAD risk SNP rs9349379 affects expression of the short *PHACTR1* transcript in macrophages**. *PHACTR1* expression in primary human macrophages relative to *GAPDH*. (A) A significant genotype-expression level correlation was demonstrated in primary human macrophages (n = 19, AA = 6, AG = 8, GG = 5). Individuals with the AA genotype had the highest level of expression of the short *PHACTR1* transcript and subjects with the GG genotype had the lowest expression. (B) There was no significant correlation with the intermediate transcript (n = 19). (C, D) Primary human CD14^+^-depleted PBMCs did not show a significant correlation between expression and genotype of rs9349379 (n = 20, AA = 7, AG = 8, GG = 5).

The rs9349379 SNP at the *PHACTR1* locus has been implicated by GWAS in the genetic risk of CAD [11], [34]. The mechanism by which different alleles of this SNP influence CAD risk is unknown. Rs9349379 is in an intronic region of *PHACTR1* and is not in high linkage disequilibrium with any coding SNPs; it may therefore affect transcriptional regulation of PHACTR1. Using transcript-specific real time quantitative PCR, we tested for an association between the alleles of rs9349379 and expression of PHACTR1 in macrophages and CD14^+^-depleted PBMC. To do this we used primary cells derived from healthy non-smoking Caucasian individuals controlled for age (>18, <50 years), body mass index, cholesterol and triglyceride levels but with differing rs9349379 genotypes. There was a significant allelic effect on expression of the short transcript; the A allele was associated with higher expression (Fig. 5A). Macrophages from individuals homozygous for the protective A allele had 2.5-fold higher expression than macrophages from individuals homozygous for the G allele, which is the coronary artery disease risk-associated allele (*p* = 0.0187). There was a trend towards reduced expression of the intermediate transcript with the A allele, but this did not reach significance (Fig. 5B). In CD14^+^-depleted PBMCs no association was detectable between genotype and expression of either the short or intermediate transcript (Fig. 5C, D). Overall, these results demonstrate that rs9349379 is both a CAD risk-associated variant and an expression quantitative trait locus (eQTL) for PHACTR1 in macrophages, but not in CD14^+^-depleted PBMC. Therefore, decreased expression of the short transcript in macrophages is associated with CAD. This genetic risk effect recapitulates the effect of LPS, which also caused suppression of the short transcript (Fig. 5A). Overall, these findings demonstrate that the CAD risk effect of genetic variants at rs9349379 may be operational in macrophages in atherosclerotic lesions.

## 4 Discussion

GWAS of complex diseases have identified genomic loci containing genetic variants that affect heritable disease risk [4]. For CAD, one of the most robust reported risk loci is at the *PHACTR1* gene locus where several studies have identified intronic SNPs that are associated with the risk of myocardial infarction, coronary stenosis and coronary calcification [9], [10], [11], [34]. The same CAD risk SNP allele is associated with protection from a non-atherosclerotic form of vascular disease; cervical artery dissection. Unravelling the biological pathways underpinning the pleiotropic effect of the *PHACTR1* locus is critical to harnessing the genetic knowledge yielded by GWAS studies [14]. To better understand the role of PHACTR1 in atherosclerosis, we investigated PHACTR1 expression and the regulatory influence of inflammatory stimuli, atherogenic lipid and genetic variation.

Using immunohistochemistry, we demonstrated increased expression of PHACTR1 in atherosclerotic lesions *in vivo*. Lipid-laden macrophages or foam cells were clearly the most abundant source of PHACTR1 in plaques. The distribution was both nuclear and cytoplasmic in macrophages, foam cells and adventitial lymphocytes, whereas in secondary lymphoid tissue lymphocytes, PHACTR1 was excluded from the nucleus. This differential intracellular localization may reflect the accumulation in atherosclerotic plaques of inflammatory lipids including lysophosphatidic acid (LPA), which activates RhoA signaling causing G- to F-actin polymerization, a mechanism known to promote PHACTR1 nuclear accumulation [35].

The previously reported *PHACTR1* transcript was isolated from brain and encodes a 580 amino acid protein [18], [19]. We only found expression of this long *PHACTR1* transcript in brain. Previous molecular and functional studies have focused on this transcript, which may limit their relevance to understanding the role of PHACTR1 beyond the nervous system. In cell types involved in atherosclerotic lesions, we found that *PHACTR1* was transcribed from a more distal TSS giving rise to the intermediate transcript. This intermediate transcript was the prevalent protein isoform in immune and endothelial cells and the encoded protein includes the 4 previously characterized actin-binding RPEL domains and NLS regions. In addition, we found transcription of a short transcript from an even more distal TSS. The encoded protein would only contain two RPEL motifs and lack the N-terminal NLS. The expression pattern of the short transcript differed markedly from the longer forms and expression was only detected in immune cells, principally macrophages and B-lymphocytes. Macrophages were notable as the only cell type with high-level expression of the intermediate and short transcript. The limited distribution in immune cells is consistent with a role for the short transcript in immunity and inflammation. We were unable to detect protein expressed from the endogenous short transcript in the cell types studied despite using an antibody raised against peptides encompassing most of its coding region. This may indicate post-transcriptional effects, rapid turnover or technical limitations of the available antibodies. A striking finding was the absence of PHACTR1 protein in VSMC.

In macrophages, oxLDL exposure caused upregulation of the short transcript and suppression of the intermediate transcript whereas lipopolysaccharide and TNFα caused upregulation of the intermediate transcript and suppression of the short transcript. These findings demonstrate that PHACTR1 expression responds differently to inflammatory stimuli and to lipid or oxidative stress pathways activated by oxLDL. The effect of the lipid stimulus was not dependent on the presence of lipoproteins since synthetic oxidized phospholipids (oxPAPC) produced a similar response to oxLDL. A non-oxidized, non-lipoprotein stimulus, comprising pure cholesterol loading did not affect expression of PHACTR1 suggesting that oxidized lipids are required.

In endothelial cells only the intermediate transcript was expressed and this was upregulated by oxLDL but not by non-oxidized, non-lipoprotein cholesterol loading. In previous studies with umbilical vein endothelial cells TNFα had no effect on PHACTR1 expression [15], [23]. However, we found that PHACTR1 was upregulated by TNFα in primary aortic arterial endothelial cells, indicating the difference between adult arterial endothelial cells and neonatal umbilical cord vein endothelial cells. These findings raise the possibility that oxLDL and TNFα could influence angiogenesis or atherosclerosis by affecting PHACTR1 expression in arterial endothelial cells. A previous study in HUVECs reported upregulation of PHACTR1 in response to the classic angiogenic factor, vascular endothelial growth factor [36].

The rs9349379 SNP is robustly associated with CAD and a fine mapping study suggested it was the likely causal variant in the region [15]. Previous eQTL studies have not taken account of the different *PHACTR1* transcripts and so have not been informative about each transcript. A large-scale eQTL study in monocytes used microarray technology with a probe that would have detected multiple *PHACTR1* transcripts [37]. An eQTL study of CAD candidate genes, did not detect significant *PHACTR1* expression in PBMC, but the Taqman assay used would only assess the long transcript, which is not expressed in these cells [38]. An eQTL for *PHACTR1* expression has recently been demonstrated in right coronary artery tissue, but the cellular origin of this effect was unclear due to the mixed cell populations in the tissue samples [15]. In addition, the different transcripts were not distinguished because the qPCR primers annealed to both the long and intermediate transcripts and the short transcript was not studied [15]. Using RNA-seq eQTLs were sought across a panel of human tissues and the rs9349379 SNP was found to be an eQTL for *PHACTR1* in coronary artery, tibial artery and aortic artery tissue; the reference allele A was associated with higher PHACTR1 expression than the alternative G allele [16]. In this study, the composite nature of the tissue samples does not allow identification of the cellular origin of the effect and the transcript affected was not reported. The direction of genetic effect is the same as that of the short transcript in the present study. An eQTL study in endothelial cells using primers that would detect only the longer transcripts did not find a significant association with variants at the rs12526453 SNP, which is in linkage disequilibrium with rs9349379 (R^2^ with rs9349379 = 0.32) [39]. We have now established a new association in macrophages between expression of the previously uncharacterized short *PHACTR1* transcript and genotype at rs9349379. In PBMCs-depleted of CD14^+^ cells (predominantly lymphocytes) there was a trend towards an eQTL effect in the opposite direction to that in macrophages. A previous study found that 4.4% of eQTL-associated SNPs produced opposite effects in different tissues [40].

Suppression of the short *PHACTR1* transcript in macrophages by pro-inflammatory stimuli mirrors the effect of the CAD risk allele (rs9349379-G), which is associated with reduced expression of the short transcript compared to that seen with the protective allele (rs9349379-A). This suggests that having the risk genotype is, in this sense, equivalent to a persistent inflammatory stimulus, such as that caused by LPS. As the short transcript was only expressed in immune cells, these data imply that the short transcript could play a role in the macrophage inflammatory response in atherosclerotic plaques.

The rs9349379 SNP is located about 250 kb upstream from the short *PHACTR1* transcript. At other risk loci chromatin looping has been established as a mechanism whereby regulatory elements containing the SNP are brought into proximity with the gene whose expression is altered. The *DEXI* gene, for example, is physically linked by chromatin looping to a type 1 diabetes risk SNP about 150 kb away and the SNP has an eQTL for *DEXI* [41]. We and others have found that GWAS risk SNPs alleles affect enhancer function by altering transcription factor binding affinity at regulatory elements [6], [17]. We hypothesize that such a mechanism may be responsible for the effect of rs9349379 and the G allele is predicted to reduce binding of MEF2 transcription factors, that are expressed in macrophages [17], [42], [43], [44].

Determining the mechanisms underlying the CAD risk conferred by GWAS-identified genetic risk variants is a major challenge for atherosclerosis research, especially for loci containing poorly characterized genes. We establish *PHACTR1* as an important gene expressed in atherosclerotic lesions and as the candidate gene at the *PHACTR1* CAD locus by demonstrating genetic regulation of a novel transcript and regulation by atherogenic stimuli. Previous molecular studies of PHACTR1 focused on a transcript which we show is not expressed in cells involved in atherosclerotic lesions. We have identified novel intermediate and short length transcripts as the dominant forms expressed in cells involved in atherosclerosis. Our genotype-expression analysis shows that the CAD-associated genetic variant is active in macrophages, the dominant cell type expressing PHACTR1 in human atherosclerotic plaques. The effects of the CAD risk genotype mirror those of an inflammatory stimulus.

## Conflict of interest

The authors declared that they do not have anything to disclose regarding conflict of interest with respect to this manuscript.

## Financial support

We thank the volunteers from the Oxford Biobank, NIHR Oxford Biomedical Research Centre, for their participation. The Oxford Biobank (www.oxfordbiobank.org.uk) is also part of the NIHR National Bioresource which supported the recalling process of the volunteers. This work was supported by the Wellcome Trust (097089/Z/11/Z), the Medical Research Council (G116/165), the Novo Nordisk Foundation (Grant Number NNF15CC0018346) and the National Institute for Health Research Oxford Comprehensive Biomedical Research Centre Program.

## Author contributions

MER and COC designed the study. MER, DL, AC and EJS performed the experiments. MER and COC wrote the manuscript. All authors analyzed the results and approved the final version of the manuscript.

## Figures and Tables

**Table 1:** Summary of expression pattern of 3 different *PHACTR1* transcripts (mRNA) in primary human cell types relevant to atherosclerosis.

	Long	Intermediate	Short
Macrophages	No	Yes	Yes
Arterial endothelial cells	No	Yes	No
Vascular smooth muscle cells	No	Yes	No
PBMC depleted of CD14^+^ cells	No	Yes	Yes
B cells (CD19^+^)	No	Barely detectable	Yes
T cells (CD3^+^)	No	No	Barely detectable
