# Supplementary material for: Genetic and environmental risk factors for atherosclerosis regulate transcription of phosphatase and actin regulating gene PHACTR1
Source: Atherosclerosis. 2016 Jul;250:95–105. doi: 10.1016/j.atherosclerosis.2016.04.025 (PMC4917897; doi:10.1016/j.atherosclerosis.2016.04.025)
Supplement: Supplementary file 1 [file mmc1.docx]

**Genetic and environmental risk factors for atherosclerosis regulate transcription of phosphatase and actin regulating gene PHACTR1**

**Michael E Reschen, Da Lin, Anil Chalisey, Elizabeth J Soilleux, Christopher A. O'Callaghan**

# SUPPLEMENTARY INFORMATION

**Supplementary Data Legend**

**Supplementary data 1.** The DNA and protein sequence of the novel ‘intermediate’ PHACTR1 transcript is shown. The sequence of 5’ RACE clones are also shown to demonstrate the transcription start site of PHACTR1 in the cell types indicated.

**Supplementary Data 1**

**Intermediate transcript – open reading frame nucleotide sequence**

ATGCGTTCTGACTCCCTCGTCCCAGGCACCCACACCCCACCCATCCGCAGGAGAAGTAAGTTTGCCAACCTGGGAAGGATTTTCAAGCCTTGGAAATGGAGGAAGAAGAAAAGCGAAAAGTTCAAACACACGTCAGCAGCCCTGGAAAGGAAAATATCTATGAGGCAAAGCAGAGAAGAGCTGATAAAGCGAGGAGTCCTGAAGGAAATCTATGATAAAGATGGGGAACTCTCTATATCCAATGAAGAGGACTCCCTAGAAAATGGGCAGTCCCTGAGCTCCAGCCAGCTGTCTCTGCCTGCCCTGTCCGAAATGGAGCCAGTCCCAATGCCCAGGGATCCCTGCTCATATGAGGTGCTCCAACCGTCAGACATCATGGATGGGCCAGTGTCTGAAGAGAGTCCCTCTGCCAGTGAGTCTGGAGTCCTCCTGTCCCAAGATCCTTCAGCCAAACCAGTCCTGCTACTGCCCCCCAAAAAACCTGCTGCTTTCCCTGGAGACCATGAAGAGACCCCAGTGAAGCAGCTGCCCCTTCTCAAGCAGCCCCCGGCCCTGCCTCCCAAACCCACTACCAGGATTGCCAACCACTTAACAGATCCTGGCGCCCCTGTGAAATTGCCTTGTCTGCCAGTGAAACTGTCGCCTCCGCTACCTCCAAAGAAAGTCATGATCTGTATGCCCGTGGGGGGGCCAGACCTCTCACTGGTGTCCTACACAGCCCAGAAGAGTGGCCAGCAGGGTGTGGCCCAGCACCACCACACTGTCCTGCCCTCCCAGATCCAGCACCAGCTGCAGTACGGCAGCCACGGCCAGCACCTCCCCTCCACCACCGGCTCCCTCCCCATGCACCCCTCGGGCTGCAGAATGATAGACGAGCTCAACAAAACGCTGGCCATGACCATGCAGAGGCTGGAAAGCTCTGAGCAGCGGGTCCCCTGTTCCACTTCTTACCACAGCTCTGGGTTGCACTCGGGTGATGGGGTCACCAAAGCAGGACCTATGGGCCTTCCAGAAATAAGACAAGTGCCAACTGTTGTGATTGAATGTGATGACAATAAAGAAAATGTGCCTCATGAGTCAGACTACGAAGACTCTTCTTGCCTGTATACAAGAGAAGAGGAGGAAGAGGAGGAGGACGAAGACGACGACAGCTCATTATACACCAGCTCCCTGGCCATGAAGGTCTGCAGGAAGGACTCCTTAGCCATCAAACTCAGCAACAGGCCCTCCAAGCGAGAGCTGGAAGAAAAGAACATCCTTCCCAGGCAGACGGATGAGGAGCGGCTGGAGCTGAGGCAACAGATTGGCACCAAGCTCACCAGGCGGCTGAGCCAGAGGCCAACTGCAGAGGAACTGGAACAGAGGAACATTTTGAAACCTCGGAATGAACAAGAGGAACAGGAGGAGAAGAGAGAGATCAAGAGGAGGCTAACCCGAAAGCTCAGTCAAAGGCCCACGGTGGAAGAGCTTCGGGAAAGAAAGATCCTCATCCGCTTCAGTGACTACGTGGAGGTGGCTGACGCTCAGGACTATGACCGCAGGGCAGATAAGCCGTGGACCCGCCTCACCGCTGCAGACAAAGCTGCCATCCGAAAGGAGCTCAATGAATTCAAAAGCACTGAGATGGAAGTTCATGAATTGAGTAGACACTTAACAAGGTTTCACCGACCTTAA

**Intermediate transcript amino acid sequence**

MRSDSLVPGTHTPPIRRRSKFANLGRIFKPWKWRKKKSEKFKHTSAALERKISMRQSREELIKRGVLKEIYDKDGELSISNEEDSLENGQSLSSSQLSLPALSEMEPVPMPRDPCSYEVLQPSDIMDGPVSEESPSASESGVLLSQDPSAKPVLLLPPKKPAAFPGDHEETPVKQLPLLKQPPALPPKPTTRIANHLTDPGAPVKLPCLPVKLSPPLPPKKVMICMPVGGPDLSLVSYTAQKSGQQGVAQHHHTVLPSQIQHQLQYGSHGQHLPSTTGSLPMHPSGCRMIDELNKTLAMTMQRLESSEQRVPCSTSYHSSGLHSGDGVTKAGPMGLPEIRQVPTVVIECDDNKENVPHESDYEDSSCLYTREEEEEEEDEDDDSSLYTSSLAMKVCRKDSLAIKLSNRPSKRELEEKNILPRQTDEERLELRQQIGTKLTRRLSQRPTAEELEQRNILKPRNEQEEQEEKREIKRRLTRKLSQRPTVEELRERKILIRFSDYVEVADAQDYDRRADKPWTRLTAADKAAIRKELNEFKSTEMEVHELSRHLTRFHRP*

**Macrophage 5’ prime RACE sequenced clone**

CTCGGCGCGAGTGTGTTGACTTTTCGCTTTTCTTCTTCCTCCATTTCCAAGGCTTGAAAATCCTTCCCAGGTTGGCAAACTTACTTCTCCTGCGGATGGGTGGGGTGTGGGTGCCTGGGACGAGGGAGTCAGAACGCATCGCCGCCAGCCTCTCCACTTCCTCAGCTTAAAGGCTGCCCAAAGCTCTTTGCTCCCACATGCATCCGCCTCTCTGGGTCTGTTTCCTGGCTTTTTAAAAAAAAAAAAAAAAAAAAAAAAAAAAAAAAAAAAAAAA

RC

TTTTTTTTTTTTTTTTTTTTTTTTTTTTTTTTTTTTTTTTAAAAAGCCAGGAAACAGACCCAGAGAGGCGGATGCATGTGGGAGCAAAGAGCTTTGGGCAGCCTTTAAGCTGAGGAAGTGGAGAGGCTGGCGGCGATGCGTTCTGACTCCCTCGTCCCAGGCACCCACACCCCACCCATCCGCAGGAGAAGTAAGTTTGCCAACCTGGGAAGGATTTTCAAGCCTTGGAAATGGAGGAAGAAGAAAAGCGAAAAGTCAACACACTCGCGCCGAG

Combined with the intermediate transcript coding sequence

TTTTTTTTTTTTTTTTTTTTTTTTTTTTTTTTTTTTTTTTAAAAAGCCAGGAAACAGACCCAGAGAGGCGGATGCATGTGGGAGCAAAGAGCTTTGGGCAGCCTTTAAGCTGAGGAAGTGGAGAGGCTGGCGGCGATGCGTTCTGACTCCCTCGTCCCAGGCACCCACACCCCACCCATCCGCAGGAGAAGTAAGTTTGCCAACCTGGGAAGGATTTTCAAGCCTTGGAAATGGAGGAAGAAGAAAAGCGAAAAGTTCAAACACACGTCAGCAGCCCTGGAAAGGAAAATATCTATGAGGCAAAGCAGAGAAGAGCTGATAAAGCGAGGAGTCCTGAAGGAAATCTATGATAAAGATGGGGAACTCTCTATATCCAATGAAGAGGACTCCCTAGAAAATGGGCAGTCCCTGAGCTCCAGCCAGCTGTCTCTGCCTGCCCTGTCCGAAATGGAGCCAGTCCCAATGCCCAGGGATCCCTGCTCATATGAGGTGCTCCAACCGTCAGACATCATGGATGGGCCAGTGTCTGAAGAGAGTCCCTCTGCCAGTGAGTCTGGAGTCCTCCTGTCCCAAGATCCTTCAGCCAAACCAGTCCTGCTACTGCCCCCCAAAAAACCTGCTGCTTTCCCTGGAGACCATGAAGAGACCCCAGTGAAGCAGCTGCCCCTTCTCAAGCAGCCCCCGGCCCTGCCTCCCAAACCCACTACCAGGATTGCCAACCACTTAACAGATCCTGGCGCCCCTGTGAAATTGCCTTGTCTGCCAGTGAAACTGTCGCCTCCGCTACCTCCAAAGAAAGTCATGATCTGTATGCCCGTGGGGGGGCCAGACCTCTCACTGGTGTCCTACACAGCCCAGAAGAGTGGCCAGCAGGGTGTGGCCCAGCACCACCACACTGTCCTGCCCTCCCAGATCCAGCACCAGCTGCAGTACGGCAGCCACGGCCAGCACCTCCCCTCCACCACCGGCTCCCTCCCCATGCACCCCTCGGGCTGCAGAATGATAGACGAGCTCAACAAAACGCTGGCCATGACCATGCAGAGGCTGGAAAGCTCTGAGCAGCGGGTCCCCTGTTCCACTTCTTACCACAGCTCTGGGTTGCACTCGGGTGATGGGGTCACCAAAGCAGGACCTATGGGCCTTCCAGAAATAAGACAAGTGCCAACTGTTGTGATTGAATGTGATGACAATAAAGAAAATGTGCCTCATGAGTCAGACTACGAAGACTCTTCTTGCCTGTATACAAGAGAAGAGGAGGAAGAGGAGGAGGACGAAGACGACGACAGCTCATTATACACCAGCTCCCTGGCCATGAAGGTCTGCAGGAAGGACTCCTTAGCCATCAAACTCAGCAACAGGCCCTCCAAGCGAGAGCTGGAAGAAAAGAACATCCTTCCCAGGCAGACGGATGAGGAGCGGCTGGAGCTGAGGCAACAGATTGGCACCAAGCTCACCAGGCGGCTGAGCCAGAGGCCAACTGCAGAGGAACTGGAACAGAGGAACATTTTGAAACCTCGGAATGAACAAGAGGAACAGGAGGAGAAGAGAGAGATCAAGAGGAGGCTAACCCGAAAGCTCAGTCAAAGGCCCACGGTGGAAGAGCTTCGGGAAAGAAAGATCCTCATCCGCTTCAGTGACTACGTGGAGGTGGCTGACGCTCAGGACTATGACCGCAGGGCAGATAAGCCGTGGACCCGCCTCACCGCTGCAGACAAAGCTGCCATCCGAAAGGAGCTCAATGAATTCAAAAGCACTGAGATGGAAGTTCATGAATTGAGTAGACACTTAACAAGGTTTCACCGACCTTAA

**Aortic endothelial cell 5’ prime RACE sequenced clone**

CGCGAGTGTGTTGACTTTTCGCTTTTCTTCTTCCTCCATTTCCAGGCTTGAAAATCCTTCCCATGTTGGCAAACTTACTTCTCCTGCGGATGGGTGGGGTGTGGGTGCCTGGGACGAGGGAGTCAGAACGCATCGCCGCCAGCCTCTCCACTTCCTCAGCTTAAAGGCTGCCCAAAGCTCTTTGCTCCCACATGCATCCGCCTCTCTGGGTCTGTTTCCACAAAAAAAAAAAAAAAA

Reverse complement:

TTTTTTTTTTTTTTTTGTGGAAACAGACCCAGAGAGGCGGATGCATGTGGGAGCAAAGAGCTTTGGGCAGCCTTTAAGCTGAGGAAGTGGAGAGGCTGGCGGCGATGCGTTCTGACTCCCTCGTCCCAGGCACCCACACCCCACCCATCCGCAGGAGAAGTAAGTTTGCCAACATGGGAAGGATTTTCAAGCCTGGAAATGGAGGAAGAAGAAAAGCGAAAAGTCAACACACTCGCG

**CD14^+^-depleted PBMC 5’ prime RACE sequenced clone**

TCGGCGCGAGTGTGTTTGACTTTTCGCTTTTCTTCTTCCTCCATTTCCAGGCTTGAAAATCCTTCCCAGGTTAGCAAACTTACTTCTCCTGCGGATGGGTGGGGTGTGGGTGCCTGGGACGAGGGAGTCAGAACGCATCGCCGCCAGCCTCTCCACTTCCTCAGCTTCACAGAGCCCCAGTCCTTAGAAGCCCAGCTGGCGTCTCCAAGGAGTCCTGCGTTTTACTTGACATTGGAGGCAACCGGTTGCAGAAACAGGGAACATTTCAAAATGAAACAGAAAGTAACCCGGGGATCCAAACCAGACCCCCGACCTGCCTCGGCCGTCCCCAATCTAGAAAAAAAAAAAAAAAA

Reverse complement:

TTTTTTTTTTTTTTTTCTAGATTGGGGACGGCCGAGGCAGGTCGGGGGTCTGGTTTGGATCCCCGGGTTACTTTCTGTTTCATTTTGAAATGTTCCCTGTTTCTGCAACCGGTTGCCTCCAATGTCAAGTAAAACGCAGGACTCCTTGGAGACGCCAGCTGGGCTTCTAAGGACTGGGGCTCTGTGAAGCTGAGGAAGTGGAGAGGCTGGCGGCGATGCGTTCTGACTCCCTCGTCCCAGGCACCCACACCCCACCCATCCGCAGGAGAAGTAAGTTTGCTAACCTGGGAAGGATTTTCAAGCCTGGAAATGGAGGAAGAAGAAAAGCGAAAAGTCAAACACACTCGCGCCGA

Combined with intermediate transcript coding sequence

TTTTTTTTTTTTTTTTCTAGATTGGGGACGGCCGAGGCAGGTCGGGGGTCTGGTTTGGATCCCCGGGTTACTTTCTGTTTCATTTTGAAATGTTCCCTGTTTCTGCAACCGGTTGCCTCCAATGTCAAGTAAAACGCAGGACTCCTTGGAGACGCCAGCTGGGCTTCTAAGGACTGGGGCTCTGTGAAGCTGAGGAAGTGGAGAGGCTGGCGGCGATGCGTTCTGACTCCCTCGTCCCAGGCACCCACACCCCACCCATCCGCAGGAGAAGTAAGTTTGCCAACCTGGGAAGGATTTTCAAGCCTTGGAAATGGAGGAAGAAGAAAAGCGAAAAGTTCAAACACACGTCAGCAGCCCTGGAAAGGAAAATATCTATGAGGCAAAGCAGAGAAGAGCTGATAAAGCGAGGAGTCCTGAAGGAAATCTATGATAAAGATGGGGAACTCTCTATATCCAATGAAGAGGACTCCCTAGAAAATGGGCAGTCCCTGAGCTCCAGCCAGCTGTCTCTGCCTGCCCTGTCCGAAATGGAGCCAGTCCCAATGCCCAGGGATCCCTGCTCATATGAGGTGCTCCAACCGTCAGACATCATGGATGGGCCAGTGTCTGAAGAGAGTCCCTCTGCCAGTGAGTCTGGAGTCCTCCTGTCCCAAGATCCTTCAGCCAAACCAGTCCTGCTACTGCCCCCCAAAAAACCTGCTGCTTTCCCTGGAGACCATGAAGAGACCCCAGTGAAGCAGCTGCCCCTTCTCAAGCAGCCCCCGGCCCTGCCTCCCAAACCCACTACCAGGATTGCCAACCACTTAACAGATCCTGGCGCCCCTGTGAAATTGCCTTGTCTGCCAGTGAAACTGTCGCCTCCGCTACCTCCAAAGAAAGTCATGATCTGTATGCCCGTGGGGGGGCCAGACCTCTCACTGGTGTCCTACACAGCCCAGAAGAGTGGCCAGCAGGGTGTGGCCCAGCACCACCACACTGTCCTGCCCTCCCAGATCCAGCACCAGCTGCAGTACGGCAGCCACGGCCAGCACCTCCCCTCCACCACCGGCTCCCTCCCCATGCACCCCTCGGGCTGCAGAATGATAGACGAGCTCAACAAAACGCTGGCCATGACCATGCAGAGGCTGGAAAGCTCTGAGCAGCGGGTCCCCTGTTCCACTTCTTACCACAGCTCTGGGTTGCACTCGGGTGATGGGGTCACCAAAGCAGGACCTATGGGCCTTCCAGAAATAAGACAAGTGCCAACTGTTGTGATTGAATGTGATGACAATAAAGAAAATGTGCCTCATGAGTCAGACTACGAAGACTCTTCTTGCCTGTATACAAGAGAAGAGGAGGAAGAGGAGGAGGACGAAGACGACGACAGCTCATTATACACCAGCTCCCTGGCCATGAAGGTCTGCAGGAAGGACTCCTTAGCCATCAAACTCAGCAACAGGCCCTCCAAGCGAGAGCTGGAAGAAAAGAACATCCTTCCCAGGCAGACGGATGAGGAGCGGCTGGAGCTGAGGCAACAGATTGGCACCAAGCTCACCAGGCGGCTGAGCCAGAGGCCAACTGCAGAGGAACTGGAACAGAGGAACATTTTGAAACCTCGGAATGAACAAGAGGAACAGGAGGAGAAGAGAGAGATCAAGAGGAGGCTAACCCGAAAGCTCAGTCAAAGGCCCACGGTGGAAGAGCTTCGGGAAAGAAAGATCCTCATCCGCTTCAGTGACTACGTGGAGGTGGCTGACGCTCAGGACTATGACCGCAGGGCAGATAAGCCGTGGACCCGCCTCACCGCTGCAGACAAAGCTGCCATCCGAAAGGAGCTCAATGAATTCAAAAGCACTGAGATGGAAGTTCATGAATTGAGTAGACACTTAACAAGGTTTCACCGACCTTAA
